# Cumulative mTBI Burden In Aging Veterans: Regional Accelerated White Matter Deterioration Identified Along Tracts

**DOI:** 10.1093/geroni/igaf122.3742

**Published:** 2025-12-31

**Authors:** Tyler Robinson, Emily Van Etten, Shira Hao, David Rothlein, William Milberg, Catherine Fortier, David Salat

**Affiliations:** Department of Veterans Affairs, Boston, Boston, Massachusetts, United States; Department of Veterans Affairs, Boston, Boston, Massachusetts, United States; Department of Veterans Affairs, Boston, Boston, Massachusetts, United States; Department of Veterans Affairs, Boston, Boston, Massachusetts, United States; Department of Veterans Affairs, Boston, Boston, Massachusetts, United States; Department of Veterans Affairs, Boston, Boston, Massachusetts, United States; Department of Veterans Affairs, Boston, Boston, Massachusetts, United States

## Abstract

Along-tract analysis of white matter (WM) aging offers a spatially specific approach to identifying effects in WM tissue with greater specificity than established tract-averaged approaches, while maintaining anatomical interpretability. As such, incorporating both whole-tract means and along-tract clusters improves our ability to identify unique and complementary patterns of effects across tracts due to differences in effect distribution and cluster thresholding in both age effects and specific WM insult such as traumatic brain injury (TBI). In this study, we seek to assess the impacts of cumulative mild traumatic brain injury (mTBI) on WM tissue microstructural degeneration in aging in a veteran sample using both whole-tract means and along-tract effect clustering via Freesurfer’s TRACULA package. Whole-tract effects were found in the splenium, bilateral ventral cingulate bundle, and bilateral optic radiation. Along-tracts analysis corroborates effects in the ventral cingulate bundle and optic radiation, with further bilateral clusters of effects in the acoustic radiation, and laterally specific clusters in the fornix, middle longitudinal fasciculus, and arcuate fasciculus. In all cases, the higher mTBI burden was associated with greater degrees of WM degeneration in advancing age as reflected by microstructural measures, suggesting mTBI is associated with accelerated age-related declines in the veteran population well after recovery and into later age. Furthermore, these findings specify anatomically founded regions of localized mTBI impacts that are not captured by whole-tract methods and contribute to the aim of improving understanding of anatomical mechanisms of mTBI impact and accuracy of targeting for future diagnosis and intervention.

